# Small-scale dissolution test screening tool to select potentially substandard and falsified (SF) medicines requiring full pharmacopoeial analysis

**DOI:** 10.1038/s41598-021-91443-5

**Published:** 2021-06-09

**Authors:** Mohammad Sofiqur Rahman, Naoko Yoshida, Hirohito Tsuboi, Yuichiro Ishii, Yoshio Akimoto, Kazuko Kimura

**Affiliations:** 1 Medi-Quality Security Institute, Graduate School of Medical Sciences, Kanazawa University, Kanazawa, 920-1192 Japan; 2 Department of Clinical Pharmacy and Healthcare Sciences, Kanazawa University, Kanazawa, 920-1192 Japan; 3 AI Hospital/Macro Signal Dynamics Research and Development Center, Institute of Medical, Pharmaceutical and Health Sciences, Kanazawa University, Kakuma-machi, Kanazawa, 920-1192 Japan

**Keywords:** Health care, Health occupations, Medical research

## Abstract

The purpose of this study was to design a convenient, small-scale dissolution test for extracting potential substandard and falsified (SF) medicines that require full pharmacopoeial analysis. The probability of metronidazole samples complying with the US Pharmacopoeia (USP) dissolution test for immediate-release tablet formulations was predicted from small-scale dissolution test results using the following criteria: (1) 95% confidence interval lower limit (95% CI_low_) of the average dissolution rate of any n = 3 of n = 24 units of each sample, and (2) average and minimum dissolution rates for any n = 3 of n = 24 units. Criteria values were optimized via bootstrap sampling with Thinkeye data-mining software. Compliant metronidazole samples in the USP first-stage and second-stage dissolution test showed complying probabilities of 99.7% and 81.0%, respectively, if the average dissolution rate of n = 3 units is equal to or greater than the monograph-specified amount of dissolved drug (Q; 85% of labeled content for metronidazole). The complying probabilities were 100.0% and 79.0%, respectively, if the average dissolution rate of n = 3 units is 91% or higher and the minimum dissolution rate is 87% or higher. Suitable compliance criteria for the small-scale dissolution test are: average dissolution rate of n = 3 units is Q + 6% or more and minimum dissolution rate is Q + 2% or more.

## Introduction

The production and distribution of substandard and falsified medicines (SFs) are increasing throughout the world^1–3^. Lower and middle-income countries (LMICs) are most affected, but developed countries are not immune^4–6^. Global statistics published by the World Health Organization (WHO) indicate that one in ten pharmaceutical products in lower and middle income countries is either substandard or falsified, but it is likely that many cases are unreported^6^. The WHO defines substandard medicines as ‘out of specification’ products, i.e., authorized medical products that fail to meet either their quality standards or specifications, or both^7^. On the other hand, falsified medicines are those that deliberately/fraudulently misrepresent their identity, composition or source^7^. Substandard medical products can be due to the lack of tools and technical capacity or sometimes intention to ensure quality standards in manufacturing, supply and distribution. Falsified medical products, in contrast, results from criminal activities, which are often enabled by inadequate regulation and governance^8^.

In this context, convenient methods to detect SF medicines are still urgently required^9–13^. Ensuring the quality of pharmaceuticals in the supply chain requires studies to establish the prevalence of SFs, as well as the development of effective analytical systems to identify and characterize SFs, and effective implementation of regulatory systems^14^. However, current laboratory approaches, such as screening and pharmacopoeial chemical tests, are laborious, expensive and time-consuming^15^. Given that the medicine stock is limited in small retail markets in local areas of LMICs, it is often difficult to collect a sufficient number of samples to carry out full pharmacopoeial testing. Additionally, collection of large amounts of samples for full pharmacopoeial testing may hinder the access of patients in need to those medicines. To overcome these limitations, several of our earlier studies in Cambodia and other countries incorporated product authentication and legitimacy verification of the manufacturer as a simple approach to seek out possible SFs^16–20^. However, the low response rates from manufacturers and medicine regulatory authorities (MRAs) make this approach problematic. Here, we describe a small-scale dissolution test based on limited sampling, designed to screen samples collected in the field in order to identify potential substandard and falsified (SF) medicines that should receive full pharmacopoeial analysis.

In our previous studies, quantity, content uniformity and dissolution tests were the major chemical analyses used to assess quality^21,22^. Among those tests, the dissolution test proved to be crucial test to define a sample’s performance, particularly in Cambodia. The purpose of this study is to develop a small-scale but statistically valid dissolution test that can be used both to provide an estimate of the prevalence of SF medicines based on predetermined acceptance criteria and to detect potential SFs that should receive full pharmacopoeial analysis.

## Methods

### Samples

Metronidazole and cimetidine were selected as candidate medicines from the Essential Drug List of Cambodia based on their stability, therapeutic importance, and ease of analysis. Samples were collected from private drug outlets in Phnom Penh, Kampong Speu, Kandal, and Takeo Province of Cambodia according to a pre-planned protocol as a part of our pharmaceutical quality survey.

### Dissolution test method

The dissolution test of the metronidazole and cimetidine samples was carried out according to the modified USP protocol using 900 mL of dissolution medium for each of the units in an NTR-VS-6P dissolution apparatus (Toyama Sangyo Co. Ltd., Osaka, Japan)^23^. The medium was prepared by adding 20 mL of 5 N hydrochloric acid made up to 1000 mL by adding distilled water. Drug release studies were performed using the USP Type I rotating basket dissolution apparatus. The paddle was set to rotate at 100 rpm for 60 min (15 min for cimetidine) and the medium temperature was maintained at 37 ± 0.50 °C. The assay was conducted by high-performance liquid chromatography (HPLC) on a Shimadzu Prominence HPLC equipped with a Shimpack C18 column (150 × 4.6 mm, 5 μm) and an ultraviolet-photodiode array detector (UV-PDA, SPD-20A/20AV Series). The flow rate of the mobile phase was adjusted to 1.0 mL/min at 30 °C (column oven). The injection volume was 10 μL. The UV detection wavelengths were set to 278 and 220 nm for metronidazole and cimetidine, respectively. The flow rate, injection volume, and detection wavelength were kept constant throughout the entire analysis.

### USP acceptance criteria

Dissolution tests were carried out for n = 21 samples of metronidazole, each consisting of n = 24 units, in accordance with the USP^23^. The Q value, i.e., the amount of dissolved active ingredient specified in the monograph, expressed as a percentage of the labeled content, is 85% for metronidazole. According to the USP indication, n = 6 dosage units were sampled and tested at stage 1. The acceptance criterion of the first stage is that the dissolution rate of each of the six units is not less than Q + 5%. If the sample fails the first stage, an additional six units are sampled and tested in the second stage. If the average dissolution rate of the n = 12 units is not less than Q, and no unit has a dissolution rate of less than Q − 15%, then the sample meets the USP requirements. If the sample fails stage 1 and stage 2, an additional n = 12 dosage units are sampled and tested. The sample meets the requirements if the average dissolution rate of the n = 24 units from all three stages is greater than or equal to Q, and no more than two units have a dissolution rate of less than Q − 15%, and no unit has a dissolution rate less than Q − 25%.

In addition, n = 17 samples of cimetidine were similarly tested to validate our developed method (see below).

### Acceptance criteria settings for small-scale dissolution test

We examined two sets of criteria for the small-scale dissolution test using n = 21 samples of metronidazole and compared the outcomes with those obtained in the USP dissolution test. To validate the devised criteria, we then applied them to n = 17 samples of cimetidine preparation.

### Sample distribution and 95% confidence interval lower limit (95% CI_low_) obtained by bootstrap resampling

**Figure 1:**
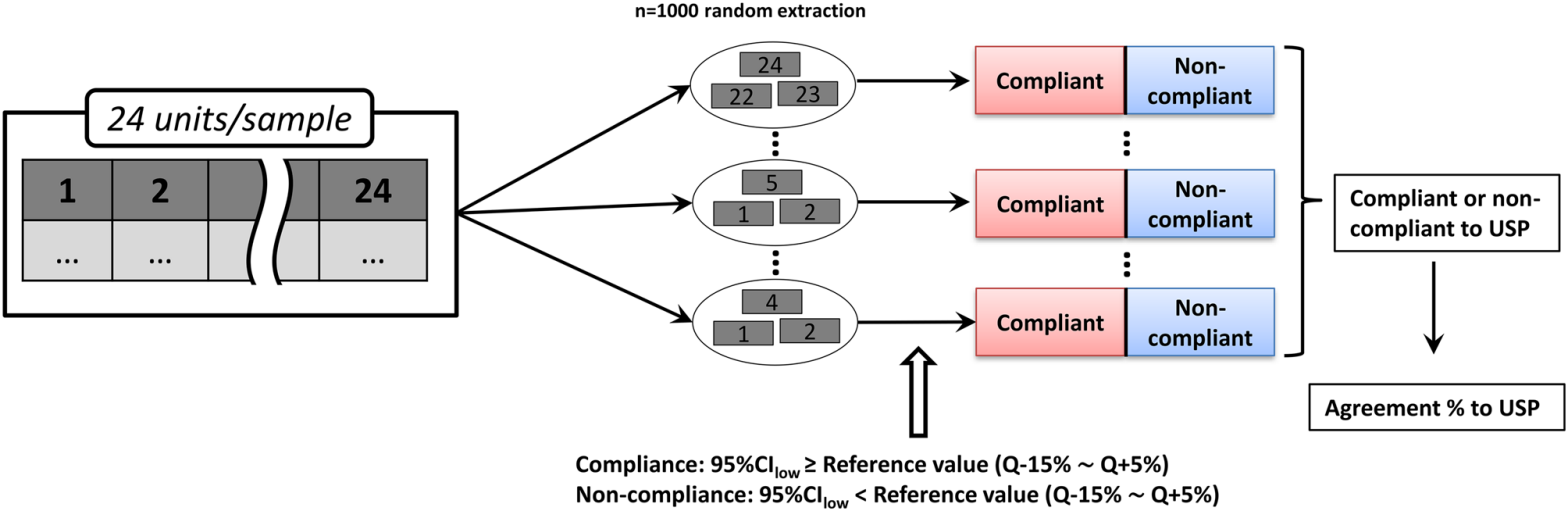
Schematic illustration of the bootstrap method based on the 95% confidence interval lower limit (95% CI_low_) as the compliance criterion.

We adopted a parametric bootstrap approach, in which multiple small data samples are randomly and repeatedly extracted from the population, to obtain the distribution of the estimated values of the sample from the mean variance^24,25^. The bootstrap method considered the extracted data (sample) as a new population and randomly resampled the data from the new population. After obtaining multiple resamples, the procedure was to judge the distribution (mean/variance) of sample estimates from these data and finally to estimate the population. In the USP dissolution test, up to n = 24 units may be tested, so all the samples examined in this procedure were resampled based on the dissolution rates of n = 24 units for each sample. Specifically, n = 3 units were randomly extracted from the n = 24 units of each sample n = 1000 times, and the lower bound of the 95% confidence interval (95% CI_low_) of the average dissolution rate was calculated (Fig. 1).

Random extraction was carried out using Microsoft Excel 2003. The criterion for reference value X to define compliance was 95% CI_low_ ≥ X% (Q + 5% ≥ X ≥ Q − 15%), and the agreement rate of each sample (the number of resamples that matched per 1000) was calculated. Since the Q value of metronidazole tablets is 85, Q + 5 is 90 and Q − 15 is 70, so we have 95% CI_low_ ≥ X% (90 ≥ X ≥ 70). By comparing the resampling results and the USP results, the reference value X that provides the best match was determined, and this was used as the compliance criterion.

### Combination of average dissolution rate and minimum dissolution rate

**Figure 2:**
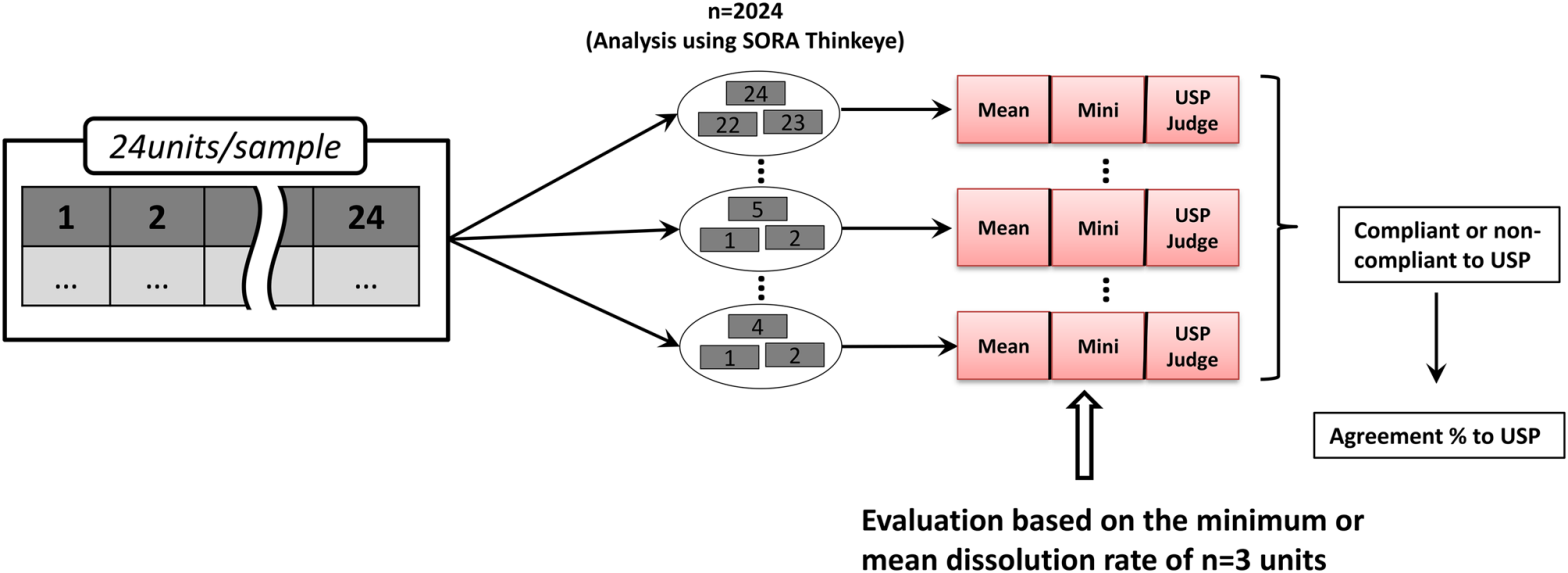
Schematic illustration of the combination of average dissolution rate and minimum dissolution rate as the compliance criterion.

**Figure 3:**
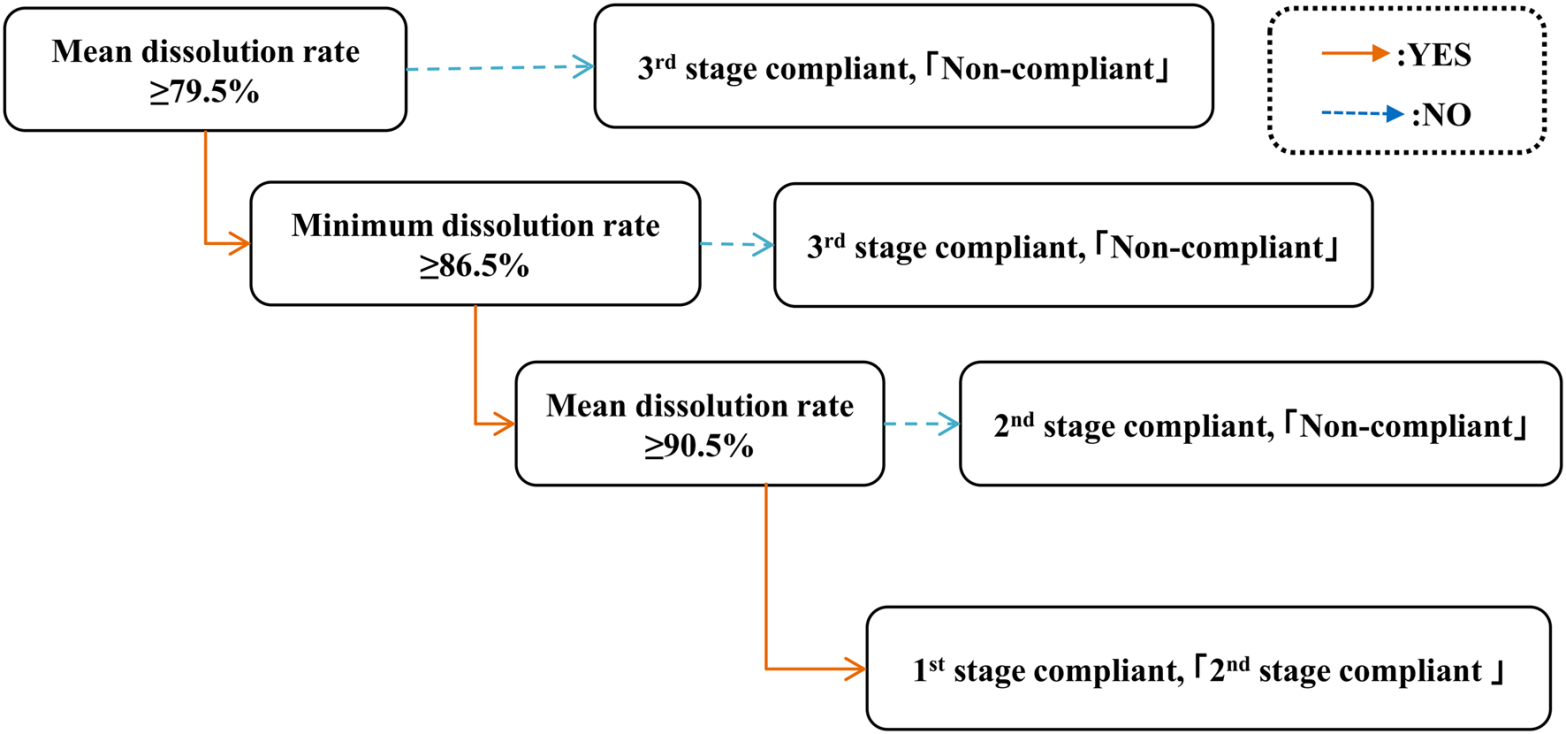
Style tree dendrogram for using the combination of average dissolution rate and minimum dissolution rate as the compliance criterion.

One thousand combinations of n = 3 of the n = 24 units were extracted (there are n = 2024 combinations in total), and the average (mean) and minimum dissolution rates were determined. These were used as independent variables and the USP result was used as a dependent variable to create a form of decision tree, known as a style tree (Figs. 2, 3). Extraction was carried out using Microsoft Excel and the process was repeated for each of the n = 21 samples.

The style tree was generated using Thinkeye software (SORA Universal Archives Inc.), which provided a dendrogram (Fig. 3) based on the Iterative Dichotomiser 3 (ID3) algorithm, using a parameter called information entropy to construct a decision tree by finding the question that best separates two or more groups^26,27^. This approach was used to examine the relationship between the dissolution rate of sets of n = 3 units and the result of USP evaluation of dissolution. In other words, it is a method of deriving the criterion that can best separate compliant and non-compliant samples. In cases where the mean and minimum values are the same but the USP judgement is different, that event was designated as “no conclusion”.

### Validation

To validate the developed methodology and criteria, they were next applied to 17 samples of cimetidine. The dissolution test of cimetidine was carried out according to USP^23^. The Q value for cimetidine is 80 and the evaluation of the dissolution test results for the samples was carried out accordingly. The lower limit of the 95% confidence interval (95% CI_low_), the minimum dissolution rate, and the average dissolution rate were determined for three units out of n = 24 units in a similar way to that described for metronidazole. Then the proposed acceptance criteria from metronidazole were applied to the cimetidine samples and compared to the USP evaluation results to evaluate the probability of the sample complying with USP dissolution test. Finally, both criteria were evaluated by comparing the respective matching rate to the USP evaluation results.

### Statistical analysis

Statistical analyses were performed using IBM SPSS statistics 19 (SPSS Inc., Chicago, IL). Confidence intervals were calculated using descriptive analysis. The criterion of significance was taken as p < 0.05. Means, standard deviations, randomizations, resamplings, etc. were calculated using SPSS 19.

## Results

### Dissolution test

Among n = 21 metronidazole samples, n = 9 were compliant in the first stage, n = 5 in the second stage, and n = 1 in the third stage, while n = 6 were non-compliant. For cimetidine preparation, n = 7 samples were compliant in first stage, n = 2 in the second stage, and n = 1 in the third stage, while n = 7 were non-compliant Details of the dissolution test for metronidazole and cimetidine samples are presented in the Supplemental Tables S1 and S2.

### Criterion value setting for 95% CI_low_ of average dissolution rates of sets of n = 3 units

**Figure 4:**
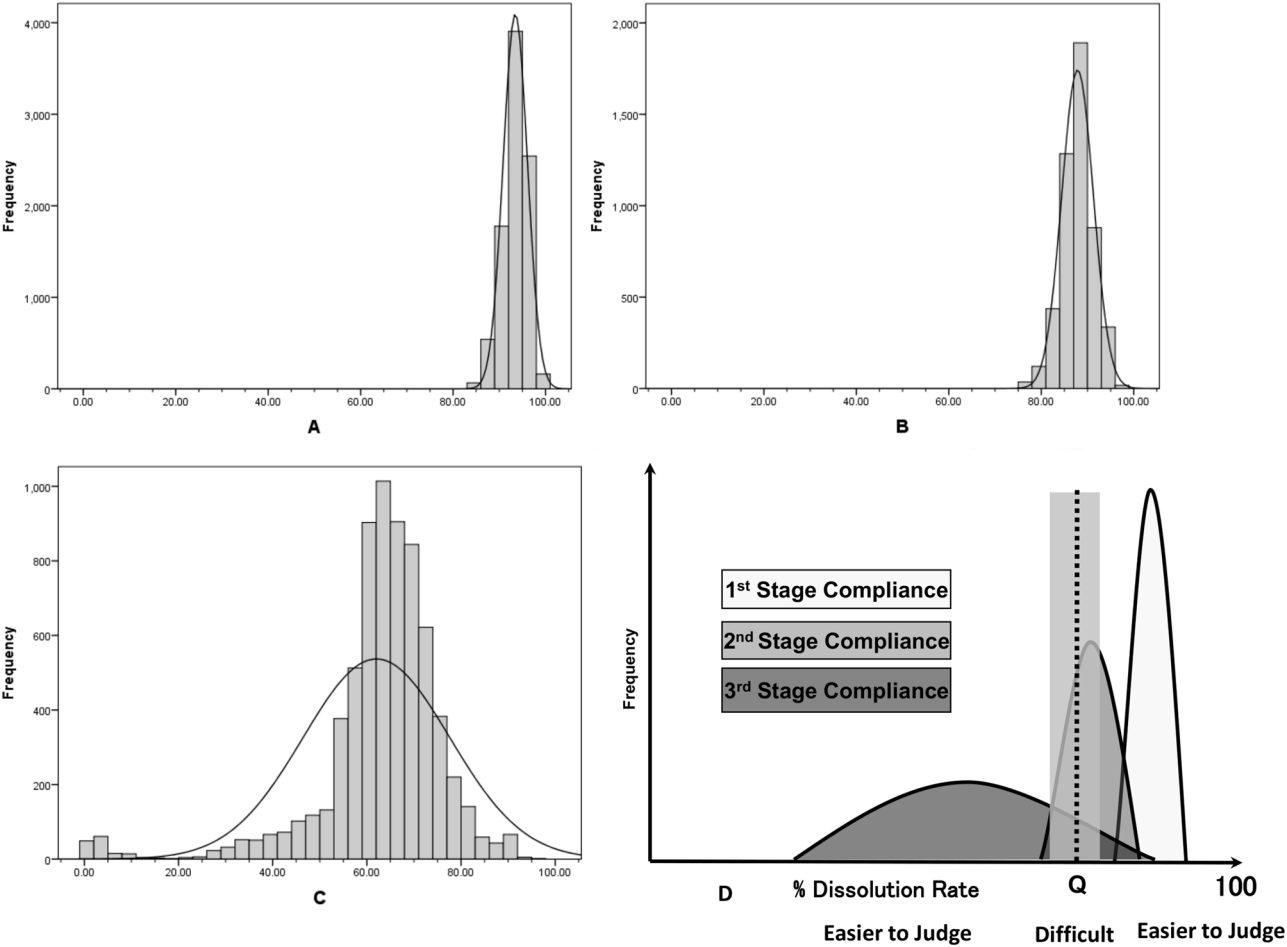
Sampling distributions for the three stages of the USP dissolution test. (**A**) First stage. (**B**) Second stage. (**C**) Third stage. (**D**) Judgement concordance rate at each stage.

The lower 95% confidence limits (95% CI_low_) of the mean dissolution rate of n = 3 units were determined with the criteria of Q − 15% to Q + 5% (Q = 85%, 70–90%) to obtain the lower bound of agreement probability with USP. The resampling process was repeated to generate tables of agreement probabilities for each of the 24 units. The agreement% for multiple virtual population means and standard deviations are presented in Table 1 and the sampling distributions obtained from 1000 bootstrap samples are illustrated in Fig. 4A–C.

**Table 1:**
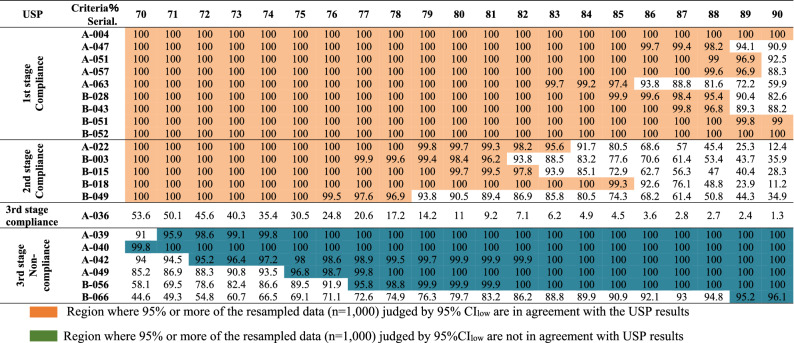
Bootstrap sampling distribution for agreement rate with USP when n = 21 samples of metronidazole were judged in terms of the 95% confidence interval lower limit value (95%CI_low_) of the average dissolution rate of n = 3 units.

According to the resampling distribution chart, if the lower 95% confidence limit of the mean dissolution rate of n = 3 units is more than 85%, the results are consistent with the first-stage USP results with a probability of more than 95%. That means there is a 95% probability that the samples will pass the first stage. Similarly, the resampling results were consistent with the USP results in the second stage with 95% probability when the 95% CI_low_ of the mean of three units was 78%. If the judgement reference was set to a lower value, higher consistency was found with USP results with higher probability. However, an anomalous result was obtained in third stage compliant sample A-036, where a mean dissolution rate of 24 units of 86.2% (close to the Q value) was obtained with 8.3% coefficient of variation (CV). For the non-compliant samples, n = 5 of n = 6 showed similar consistency with USP results if the 95% CI_low_ of the mean dissolution rate of n = 3 units was set to 77%. As the reference value is increased, the probability of agreement with USP results increases. However, the rate of agreement with the USP result for the remaining one sample (B-066) was low. The mean dissolution rate of the n = 24 units of the sample was near the Q value (84.0%) with 17.6% CV.

Thus, if the lower 95% confidence level of the mean dissolution rate of n = 3 units is 85% (equivalent to the Q value) or more, the results of the first-stage USP dissolution test can be extracted with a probability of 95% or more. On the other hand, for the samples that were compliant in the second stage the agreement rate was 70%. For the sample that was compliant in the third-stage USP dissolution test, the possibility of compliance was even lower (Fig. 4D). Among the non-compliant samples, n = 5 showed 100% agreement with USP result and the other showed 90.9% agreement. This result indicates that if the mean dissolution rate falls below the Q value, or lies close to it (for example A-0363), there is a possibility that the sample will be non-compliant. Samples showing such characteristics would require more than three units to be evaluated.

### Criterion value setting for average dissolution rate and minimum dissolution rate

First-stage dissolution test compliant samples were in 100% agreement when the average and minimum values were set to ≥ 90.5% and ≥ 86.5%, respectively. With the same criteria, it was observed that second stage compliant samples could also be extracted to some extent in addition to first stage compliant samples. As for the second-stage compliant samples, if the judgment criteria are set to a mean dissolution rate of 79.5% or more and minimum dissolution rate of 86.5% or more, non-compliant samples may also be extracted. Furthermore, it was difficult to extract third-stage compliant samples based on average dissolution rate and minimum dissolution rate of n = 3 units. Thus, it was concluded that by using the criteria that the n = 3 units average dissolution rate is 79.5% or more, the average dissolution rate is 90.5% or more, and the minimum dissolution rate is 86.5% or more, it is possible to extract first stage and second stage compliant samples without extracting non-compliant samples. For practical convenience, we decided to round the criterion values to the nearest integer using the rounding rule, as shown in Table 2. Table 3 shows the agreement rate with USP when these four criteria are applied to the n = 21 metronidazole samples.

**Table 2:** Agreement rate with USP for n = 21 samples of metronidazole judged from average dissolution rate and minimum dissolution rate of n = 3 units.

1st stage compliant samples	Agreement rate with USP results (%)				
Extraction criteria	1st stage compliant	2nd stage compliant	3rd stage compliant	Non-compliant n = 6 samples	
	n = 9 samples	n = 5 samples	n = 1 sample	n = 5 samples	n = 1 sample
Average dissolution rate ≧ 91 and minimum ≧ 87	100 ± 0	78.8 ± 23	5.29	100 ± 0	90.1
Mean ≧ 90 and minimum ≧ 87	100 ± 0	93.9 ± 5.6	5.78	100 ± 0	89.3
Mean ≧ 91 and minimum ≧ 86	100 ± 0	80.8 ± 24	6.32	100 ± 0	88.1
Mean ≧ 90 and minimum ≧ 86	100 ± 0	96.2 ± 4.7	7.36	100 ± 0	84.4

**Table 3:** Agreement rate with USP when n = 21 samples of metronidazole were judged according to the four different extraction criteria.

Criteria	Concordance rate with USP rating (%)				
	1st stage compliant	2nd stage compliant	3rd stage compliant	Non-compliant n = 6 samples	
	n = 9 samples	n = 5 samples	n = 1 sample	n = 5 samples	n = 1 sample
(A) The average dissolution rate of 3 tablets is 90% or more minimum elution rate of 86% or more	100 ± 0	96.2 ± 4.7	7.36	100 ± 0	84.4
(B) The average dissolution rate of 3 tablets is 91% or more minimum elution rate of 86% or more	100 ± 0	80.8 ± 24	6.32	100 ± 0	88.1
(C) The average dissolution rate of 3 tablets is 90% or more Minimum elution rate of 87% or more	100 ± 0	93.9 ± 5.6	5.78	100 ± 0	89.3
D) The average dissolution rate of 3 tablets is 91% or more minimum elution rate of 87% or more	100 ± 0	78.8 ± 23	5.29	100 ± 0	90.1

When comparing the above criteria, we focused on the following two points- to reliably match samples with extremely good dissolution properties such as first stage compliant samples, and to extract non-compliant samples as efficiently as possible after extracting compliant samples. Notably, 100% agreement with the USP evaluation in the first stage was obtained using any of the four criteria. For second-stage compliance, the best match with the USP evaluation was obtained using criterion A, which gave an agreement rate of 96.2%. On the other hand, criterion D gave the lowest agreement rate of 78.8%. For third-stage compliance, the best match with the USP evaluation was obtained using criterion A (7.36%), while criterion D had the lowest agreement rate (5.29%). For non-compliant samples, the best match with the USP evaluation was obtained using criterion D (90.1%), while criterion A showed the lowest agreement rate (84.4%). Since our priority in developing this simple screening method is to maximize the ability to detect non-compliant samples, we finally selected the following criteria for compliant samples- average dissolution rate of n = 3 units of Q + 6% or more and minimum dissolution rate in 3 units of Q + 2% or more.

### Validation of judgment criteria

To examine the general applicability of the selected criteria, we next applied them to a set of n = 17 cimetidine samples. The results are shown in Table 4.

**Table 4:** Agreement rate with USP for cimetidine using the two types of selected criteria in the small-scale dissolution test.

Criteria	Concordance rate with USP rating (%)					
	1st stage compliant n = 7 samples		2nd stage compliant	3rd stage compliant	Non-compliant n = 7 samples	
	n = 6 samples	n = 1 sample	n = 2 samples	n = 1 sample	n = 6 samples	n = 1 sample
1. 95% CI_low_ of 3 tablets average dissolution rate is Q value or more	100 ± 0	91.4	41.6 ± 50	5.53	100 ± 0	99.8
2. Average dissolution rate of 3 tablets is Q value + 6% or more and all dissolution rates are Q value + 2%	100 ± 0	100	23.6 ± 23	5.34	100 ± 0	98.3

As for the first stage compliant samples, when the first criterion is applied, that is if the lower 95% confidence level of the mean of n = 3 units’ average dissolution rate is the Q value or more, n = 1 sample out of n = 6 samples showed 91.4% probability of agreement with USP results and the rest were 100% consistent. When the second criterion is applied, that is the average dissolution rate of 3 units was Q value + 6% or more and all units’ dissolution rates were Q value + 2% or more, all the samples were in 100% agreement with USP results. Therefore, for the 1st stage compliant samples, the second criterion showed a higher agreement rate than the first criterion. For the second stage compliant samples, if 95% CI_low_ of the average dissolution rate of n = 3 units was equal to Q value or more, it was in agreement with USP with a probability of 41.6% on average. However, the second criterion showed an average 23% agreement with USP results. For the third stage compliant sample, both criteria showed low agreement rates—5.53 and 5.34%.

In the case of non-compliant samples, adopting criterion 1 (95% CI_low_) showed 100% agreement with USP results for n = 6 samples, with the other one showing 99.8%. With criterion 2, one sample showed 98.3% agreement and the other five showed 100% agreement with USP results. As shown in Table 4, the second criterion showed a higher agreement rate than the first criterion for the first-stage compliant samples, whereas the first criterion gave a higher agreement rate for the second-stage compliant samples. Both criteria gave poor results for third-stage compliant samples. Importantly, in the case of non-compliant samples, both criteria showed high compliance rates with the USP results (98.3–100%). Combining both the metronidazole samples and the cimetidine samples, it appears that criterion 2 is more suitable. These results indicate that the small-scale dissolution test for screening compliant samples is applicable to cimetidine as well as metronidazole.

## Discussion

Facilities for quality tests of pharmaceutical products are often limited in developing countries. Therefore, there is a need for simple, inexpensive tests to evaluate quality. In our previous studies to detect SFs, we have found that the test with the highest nonconformity rate was the dissolution test^16–20^. Therefore, we focused here on developing a small-scale dissolution test that would be able to predict the outcome of pharmacopoeial dissolution tests using a small number of samples (n = 3, as compared with n = 6 for the USP first-stage test). The method may also be adapted as part of voluntary pharmacovigilance for the pharmaceutical quality of the distributed medicines by the governments of developing countries. In addition, among n = 42 samples of metronidazole (of which n = 21 were used for the present study), n = 30 (71.4%) met the USP first-stage acceptance criteria (Supplemental Table S1). Therefore, even if the small-scale test method could not predict the outcome for samples that progress to the second or third stage of the USP test, it could significantly reduce the cost and time required for the full test by extracting samples that would be compliant at the first stage.

To leverage the data obtained with three-unit sampling, we applied bootstrap resampling methodology. We carried out 1000 random resamplings of n = 3 of the n = 24 units (there are 2024 combinations in total), as we considered this would give sufficient coverage. However, in cases where there is a large variation in dissolution rate among the n = 24 units in a sample, the CV value of the dissolution rate can be quite large (e.g., 17.6% for sample B-066), and some of the n = 24 units will show a dissolution rate of more than the Q value. In such cases, some selections of three units from different samples may have the same average and minimum dissolution values even if one sample is compliant and the other is not. As regards the criteria examined here, the 95% CI_low_ is expected to pick up samples with a low average dissolution rate as well as those with a large variation in the dissolution rate (Table 1).

For the second criterion, we used a combination of average dissolution rate and minimum dissolution rate. The use of average dissolution rate alone would be insufficient to distinguish compliant samples from non-compliant samples with a high average dissolution rate, such as B-066 (Supplemental Table S1). On the other hand, the minimum dissolution rate can pick up such samples. Finally, we found that the combination of an average dissolution rate of Q + 6% or more and a minimum dissolution rate of Q + 2% or more could identify samples that passed the USP first stage test with 100% probability. One sample (B-066) that failed the USP test had a 1.7% chance of meeting the above acceptance criteria (Table 1, Supplemental Table S1), because the dissolution rate of this sample varied greatly over the range of 37.8–97.2% (CV = 17.6%). This suggests that the non-compliant sample may show a large variation in the dissolution rate between brands as well as a large variation in the formulation within the lot (same batch number). This, further implies that with unstable manufacturing process, the lot properties cannot be predicted as some selections of three units from different samples may have the same average and minimum dissolution values even if one sample is compliant and the other is not. In such case, if the variation in the dissolution rate of the drug product is unknown, evaluation using 95% CI_low_, which can also account for the variation, is more suitable than the evaluation using the average value. Such a large variation limits the accuracy of judgments based on analysis of n = 3 units. Nevertheless, the small-scale screening method should be useful in quality surveys in the countries where only small numbers of samples can be collected from individual drug stores.

### Limitation of the study

In the present study, the small-scale dissolution method based on examination of n = 3 units of each sample was developed using n = 21 metronidazole samples and validated using n = 17 cimetidine samples. Further studies with larger numbers of samples and with other medicines will be needed to further confirm the validity of this approach and to assess its general applicability.

## Conclusion

We have developed a small-scale dissolution test using only n = 3 units per sample to predict whether samples would pass the USP dissolution test, which requires n = 6 units even for the first stage. We show that our methodology can identify samples that would pass the USP first-stage test with 100% probability using the following compliance criteria: the average dissolution rate of n = 3 units is Q + 6% or more and the minimum dissolution rate is Q + 2% or more. Such samples can thus be eliminated from further consideration, making it easier to identify potential SF medicines that should receive full pharmacopoeial analysis. This methodology should reduce the cost and time required for testing, which would be especially advantageous in developing countries.

## Supplementary Information

Supplementary Information.
