# Supplementary material for: Small-scale dissolution test screening tool to select potentially substandard and falsified (SF) medicines requiring full pharmacopoeial analysis
Source: Sci Rep. 2021 Jun 9;11:12145. doi: 10.1038/s41598-021-91443-5 (PMC8190288; doi:10.1038/s41598-021-91443-5)
Supplement: Supplementary file 1 — Supplementary Information. [file 41598_2021_91443_MOESM1_ESM.pdf]

Small-scale dissolution test screening tool to select potentially substandard and falsified (SF) medicines requiring full pharmacopoeial analysis

Mohammad Sofiqur Rahman¶*, Naoko Yoshida¶, Hirohito Tsuboi*, Yuichiro Ishii, Yoshio Akimoto, Kazuko Kimura

Supplemental Table 1. Results of small-scale dissolution test for metronidazole

Metronidazole for small scale method: Result of dissolution test (n=21)

No.	Serial .	Judge (USP)	1st stage						2nd stage						3rd stage												Mean 1−24	SD 1−24	CV% 1−24	Min value	Max value	95%CI lower	95%CI upper
			1	2	3	4	5	6	7	8	9	10	11	12	13	14	15	16	17	18	19	20	21	22	23	24							
1	A−004	○	96.4	95.7	95.2	95.8	95.0	95.5	97.5	98.1	98.4	98.2	97.5	98.7	96.7	98.3	96.2	97.1	96.3	98.5	97.9	97.1	96.9	98.4	97.1	95.2	97.0	1.19	1.23	95.0	98.7	96.5	97.5
2	A−047	○	95.1	98.9	94.7	97.7	99.7	97.2	101.0	99.8	100.4	98.9	102.9	100.5	99.6	103.4	103.0	100.6	98.7	100.5	97.4	96.0	104.9	96.7	99.3	96.8	99.3	2.62	2.63	94.7	104.9	98.2	100.4
3	A−051	○	97.0	95.9	96.5	100.0	97.8	96.2	98.0	97.6	98.5	96.4	98.8	97.0	95.9	97.9	98.9	99.5	96.4	98.6	104.0	103.8	100.4	98.2	101.3	101.7	98.6	2.30	2.34	95.9	104.0	97.6	99.6
4	A−057	○	94.8	93.3	94.5	94.6	93.4	95.3	95.9	96.8	94.5	94.4	96.1	94.1	92.8	93.8	94.7	92.7	93.3	96.0	94.6	93.0	93.5	97.7	95.5	97.0	94.7	1.37	1.45	92.7	97.7	94.1	95.3
5	A−063	○	97.0	92.1	93.7	93.1	94.1	94.4	101.6	100.0	95.2	98.3	102.1	97.3	95.0	95.1	96.8	97.6	99.9	94.8	96.8	95.2	93.4	97.4	96.4	95.1	96.3	2.62	2.71	92.1	102.1	95.2	97.5
6	B−028	○	95.6	94.7	95.3	95.2	95.5	93.9	96.9	98.4	96.9	101.9	99.3	96.5	97.0	99.3	97.0	100.4	97.5	98.1	102.8	98.4	98.7	101.9	97.7	103.4	98.0	2.57	2.62	93.9	103.4	96.9	99.1
7	B−043	○	97.4	96.6	97.1	97.5	95.5	92.2	96.0	96.4	97.0	96.5	95.4	96.7	97.2	98.4	95.5	98.5	98.8	97.1	96.9	97.7	97.6	97.9	97.2	96.4	96.8	1.34	1.39	92.2	98.8	96.2	97.4
8	B−051	○	98.2	95.6	94.6	95.8	97.9	96.2	97.5	97.9	98.8	97.9	101.0	96.8	98.5	96.7	98.1	97.5	97.3	97.6	98.2	97.8	97.7	99.9	98.2	99.6	97.7	1.39	1.42	94.6	101.0	97.1	98.3
9	B−052	○	98.8	100.2	98.3	96.9	100.8	99.1	96.3	99.0	98.3	96.9	98.7	98.3	96.2	97.4	96.6	96.4	97.9	98.8	96.2	97.7	96.1	96.2	97.3	96.9	97.7	1.32	1.35	96.1	100.8	97.2	98.3
10	A−022	○2nd	92.4	88.7	91.6	89.9	88.3	92.2	91.7	90.0	87.8	90.0	90.6	92.3	90.9	96.5	92.7	91.8	92.2	92.1	94.9	93.3	90.2	91.9	90.8	91.5	91.4	1.94	2.12	87.8	96.5	90.6	92.2
11	B−003	○2nd	96.0	91.5	88.4	91.7	92.6	93.6	97.7	101.2	96.7	94.5	95.8	89.8	94.6	95.4	95.9	95.7	96.5	93.6	94.2	95.6	99.3	95.8	90.5	93.5	94.6	2.93	3.10	88.4	101.2	93.4	95.8
12	B−015	○2nd	94.5	89.3	89.7	92.7	96.4	94.2	92.9	95.3	96.5	89.7	97.6	91.4	97.1	94.9	93.3	92.4	96.7	92.7	88.6	95.1	91.2	95.2	96.3	96.1	93.7	2.68	2.85	88.6	97.6	92.6	94.9
13	B−018	○2nd	89.7	89.5	89.6	90.1	91.2	90.9	88.7	89.3	89.3	89.6	90.6	90.4	91.2	92.2	92.4	90.2	91.1	91.6	91.9	92.9	92.6	92.6	92.6	91.8	90.9	1.29	1.42	88.7	92.9	90.4	91.5
14	B−049	○2nd	94.3	89.4	94.0	94.2	91.2	95.5	95.5	90.7	92.5	92.9	94.4	96.8	98.0	95.8	97.7	99.6	97.1	95.8	93.1	86.5	100.0	96.8	96.6	92.1	94.6	3.21	3.39	86.5	100.0	93.2	96.0
15	A−036	○3rd	77.7	77.9	78.3	82.6	80.1	83.8	85.0	85.3	86.4	85.4	96.3	94.5	91.0	94.3	92.9	80.1	71.3	89.9	92.2	79.5	84.1	100.4	93.6	87.1	86.2	7.18	8.32	71.3	100.4	83.2	89.3
16	A−039	×	69.6	77.2	71.6	73.5	72.1	68.8	68.1	72.3	71.9	74.6	74.7	73.7	68.3	65.9	68.9	66.9	73.9	72.0	71.9	68.9	70.0	71.2	64.0	72.3	70.9	3.08	4.35	64.0	77.2	69.6	72.2
17	A−040	×	64.7	61.6	70.9	67.3	66.7	65.7	68.0	66.6	64.0	69.6	70.5	69.5	65.3	69.2	68.5	68.1	70.8	64.7	66.9	64.4	66.8	65.3	64.1	66.8	66.9	2.43	3.63	61.6	70.9	65.9	68.0
18	A−042	×	75.7	55.6	66.9	69.9	77.6	64.2	76.3	69.4	88.6	80.1	60.7	52.3	61.5	72.7	76.2	84.9	64.0	65.0	82.1	80.9	62.3	87.0	68.4	80.8	71.8	9.96	13.88	52.3	88.6	67.6	76.0
19	A−049	×	72.3	67.5	74.5	75.6	76.0	77.0	66.9	77.5	65.0	74.5	72.3	77.6	77.8	72.3	75.3	71.2	71.2	68.3	64.5	78.8	76.4	66.2	76.7	75.8	73.0	4.43	6.07	64.5	78.8	71.1	74.8
20	B−056	×	73.7	79.1	72.4	74.2	70.1	76.5	81.5	72.3	79.5	75.0	78.4	74.2	78.8	78.8	77.5	81.9	78.2	74.0	73.8	78.8	77.4	85.4	74.8	78.0	76.8	3.53	4.59	70.1	85.4	75.4	78.3
21	B−066	×	86.7	80.2	81.3	41.1	91.9	81.9	92.1	86.9	88.4	92.7	97.2	76.2	93.9	85.5	81.2	37.8	89.5	93.5	91.7	85.7	93.3	80.8	91.6	94.4	84.0	14.79	17.61	37.8	97.2	77.7	90.2

○ = **Compliant**
× = **Non-compliant**
○2nd = **Compliant in 2nd Stage**
○3rd = **Compliant in 3rd Stage**

Supplemental Table 2. Results of small-scale dissolution test for cimetidine

Cimetidine for small scale method: Result of dissolution test (n=17)

No.	Serial .	Judge (USP)	1st stage						2nd stage						3rd stage												Mean 1−24	SD 1−24	CV% 1−24	Min value	Max value	95%CI lower	95%CI upper
			1	2	3	4	5	6	7	8	9	10	11	12	13	14	15	16	17	18	19	20	21	22	23	24							
1	A−002−2	○	101.3	100.9	102.8	102.7	100.4	101.4	95.2	95.3	97.0	97.2	97.1	96.4	100.3	99.9	98.8	100.6	99.2	98.8	97.9	98.2	99.5	99.2	99.2	100.1	99.1	2.07	2.09	95.2	102.8	98.3	100.0
2	A−019	○	98.8	100.5	101.0	96.3	100.6	102.7	94.5	94.7	94.7	94.1	94.9	95.8	95.3	96.7	96.5	95.0	95.9	95.4	95.7	94.3	93.8	92.8	97.1	95.1	96.3	2.55	2.65	92.8	102.7	95.3	97.4
3	A−034	○	97.2	88.1	95.0	95.0	91.9	92.2	90.5	92.2	93.6	87.5	94.2	94.7	95.0	90.0	92.2	90.6	92.7	92.4	92.9	94.7	92.1	91.6	90.7	85.4	92.2	2.68	2.91	85.4	97.2	91.0	93.3
4	A−058	○	103.1	101.6	100.5	101.4	101.3	101.6	98.3	97.7	97.1	98.6	98.0	97.3	97.1	99.1	97.8	97.0	98.9	99.5	95.6	98.2	98.4	98.5	95.5	101.6	98.9	2.03	2.06	95.5	103.1	98.0	99.8
5	A−069	○	99.2	101.5	95.5	97.5	101.5	95.6	101.2	96.5	95.8	95.2	95.1	95.3	94.5	99.0	92.7	91.3	94.9	96.4	96.5	96.5	95.2	94.7	96.4	95.6	96.4	2.53	2.63	91.3	101.5	95.3	97.5
6	B−012−2	○	99.7	99.8	99.8	99.1	97.6	98.8	97.2	95.3	97.5	97.6	96.4	95.9	97.3	97.7	96.0	94.1	97.0	97.3	94.6	95.8	95.2	96.8	98.5	96.7	97.1	1.60	1.65	94.1	99.8	96.5	97.8
7	B−023	○	94.6	93.1	97.1	98.3	100.7	98.6	97.6	96.5	95.7	96.6	96.8	96.7	96.9	95.8	95.6	97.0	95.8	96.0	94.2	94.7	96.4	94.4	96.2	96.7	96.3	1.58	1.64	93.1	100.7	95.7	97.0
8	A−054	○2nd	80.4	85.4	83.5	82.8	85.9	86.1	87.1	88.3	85.1	84.0	83.9	87.5	84.6	84.5	87.7	88.3	87.3	86.8	84.6	86.1	86.3	85.2	87.5	86.9	85.7	1.91	2.23	80.4	88.3	84.8	86.5
9	B−022	○2nd	78.6	82.6	76.6	85.7	86.0	81.5	84.7	80.1	86.7	84.9	87.7	82.3	82.0	68.0	86.5	83.9	84.6	72.8	66.8	70.4	66.9	92.2	96.2	75.4	81.0	7.73	9.54	66.8	96.2	77.7	84.2
10	B−078	○3rd	79.5	64.8	76.9	75.8	69.9	75.1	88.7	89.2	87.7	85.4	89.6	85.9	74.5	80.1	84.1	81.0	88.8	79.8	72.1	78.2	81.6	80.6	88.0	84.8	80.9	6.66	8.23	64.8	89.6	78.1	83.7
11	A−014	×	3.2	3.1	2.2	2.3	2.5	2.7	4.7	4.1	6.8	6.2	5.9	4.6	3.7	4.1	28.3	5.1	5.9	4.5	5.4	5.9	6.3	6.4	5.4	5.2	5.6	5.04	89.88	2.2	28.3	3.5	7.7
12	A−020−2	×	31.6	30.6	32.9	33.2	38.5	36.1	23.8	29.5	24.4	27.3	28.7	30.4	29.6	28.7	30.1	27.4	27.4	19.4	24.0	33.7	24.2	26.5	30.4	24.8	28.9	4.33	14.98	19.4	38.5	27.1	30.7
13	A−075	×	73.8	69.7	73.5	75.6	70.0	72.8	66.4	69.8	73.7	83.1	74.5	77.6	69.8	76.5	67.2	62.1	71.3	75.1	62.4	64.0	69.3	76.0	67.4	69.7	71.3	5.02	7.04	62.1	83.1	69.2	73.4
14	A−076	×	101.6	25.8	96.4	16.4	34.5	103.4	14.1	21.1	85.1	31.1	22.5	90.5	28.2	92.7	70.5	84.5	21.5	21.0	8.7	7.8	6.5	8.3	7.1	7.4	42.0	36.42	86.83	6.5	103.4	26.6	57.3
15	B−044	×	12.6	5.4	5.3	11.4	3.1	9.1	4.5	7.8	9.9	5.7	4.9	9.1	4.7	5.6	9.4	4.6	8.3	4.7	8.7	7.8	6.5	8.3	7.1	7.4	7.2	2.40	33.47	3.1	12.6	6.1	8.2
16	B−077	×	81.8	76.3	79.9	75.1	79.4	77.9	74.3	74.3	74.1	75.3	74.5	78.0	74.6	75.4	73.7	74.2	74.6	74.1	74.1	71.5	71.6	72.2	75.1	74.6	75.3	2.51	3.34	71.5	81.8	74.2	76.3
17	PP−001	×	71.1	72.9	73.1	72.7	74.0	73.3	74.6	73.5	74.2	76.0	74.7	77.3	80.1	80.7	79.7	77.0	78.4	78.2	79.9	78.4	75.2	77.2	79.9	78.3	76.3	2.83	3.72	71.1	80.7	75.1	77.5

○ = **Compliant**
× = **Non-compliant**
○2nd = **Compliant in 2nd Stage**
○3rd = **Compliant in 3rd Stage**
